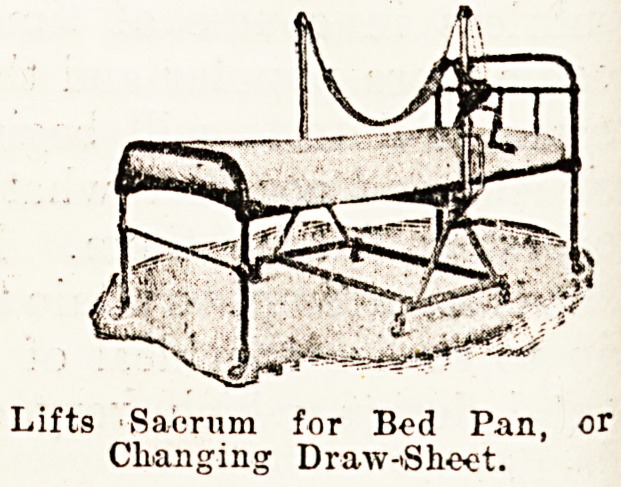# The Latest Types of Invalid Lifter

**Published:** 1912-04-27

**Authors:** 


### The Latest Types of Invalid Lifter.

There is one exhibit at the Ideal Home Exhibition which should claim hospital workers' attention. This is the exhibit of Mr. Skeffington's patent invalid lifters, practical demonstrations of which are being daily given. The mechanical devices for raising heavy or helpless patients which have been invented by Mr. Skeffington are in the first place efficient for the several purposes for which they are intended, and in the second place are

The Skeffiington Lifts for Making Bed Underneath.

For Appendicitis, Abdominal Operations and Maternity.

capable of that adaptability to varying conditions which is indispensable if they are to prove a boon to the medical profession, nurses, and to the sick.

The Skeffington is an apparatus which has already been brought before the notice of the profession, and which is in use at the present time in several parts of the country. By means of an easily controlled mechanism governing two rollers placed at either end of the bed, a patient can be raised with the expenditure of very little energy on the part of the nurse. The changing of sheets, the making of the bed, or the manipulation of the bed-pan becomes a matter of no difficulty or strain, even with the heaviest patient.

The patent sacrum lifter is another piece of apparatus for which a large amount of success may be predicted. This may be had in two types—the mobile form, which before use is folded down and run in under the bed, and the portable type, the framework of which is in two parts, which stand one on either side of the bed. With this lifter one person can easily lift a heavy patient up for any purpose, or it can be so adjusted as to turn him on one side and to maintain him in that position.

Another extremely simple lifter is the patent lifting cushion, which consists of a rubber bag of special design to be easily drawn under a patient; this is then inflated by means of an air-pump, when it will gently lift the patient's sacrum the necessary few inches for the easy use of the bed-pan.

The Skeffington inclinator is a more recent product, and should appeal strongly to hospital surgeons. It consists of a steel mattress frame which is extraordinarily light, and can be carried easily by any nurse. This is pushed into position under the bedding, and then by the aid of two specially designed three-wheel pulleys hooked to a steel pole at the head of the bed, a patient can be comfortably and with ease tilted into the required position. As an efficient means of obtaining the Fowler position, this simple inclinator is superior, more comfortable, and more stable than any method we have come across. One more of Mr. Skeffington's inventions for the comfort of invalids and the saving of labour we must draw attention to, and that is the dysentery mattress, which is characterised by its central collapsible section. This section can be adjusted by inflation with air or by a simple mechanical apparatus, which by the turning of a handle fills up the gap between the two other sections of the mattress. The collapsible section can be withdrawn in a

Mobile Type. Wheels under the Bed.

Lifts Sacrum for Bed Pan, or Changing Draw-Sheet.

moment, allowing the draw-sheet to be easily changed or the bed-pan placed in position in the cavity left.

We have perhaps indicated sufficiently by the above examples the type and the usefulness of the apparatus to be seen in working order and subjected to practical tests at Skeffington's stall at Olympia.

## Figures and Tables

**Figure f1:**
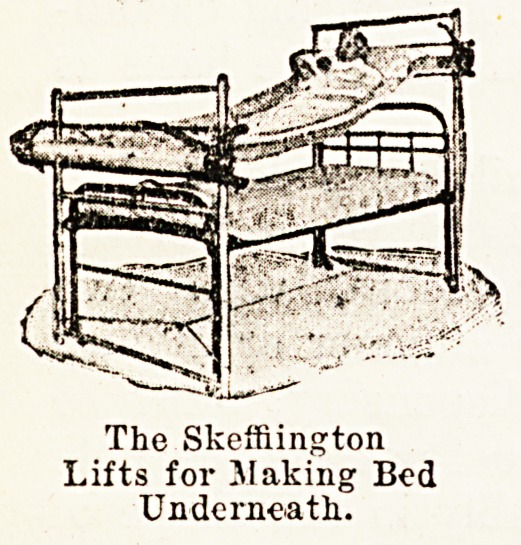


**Figure f2:**
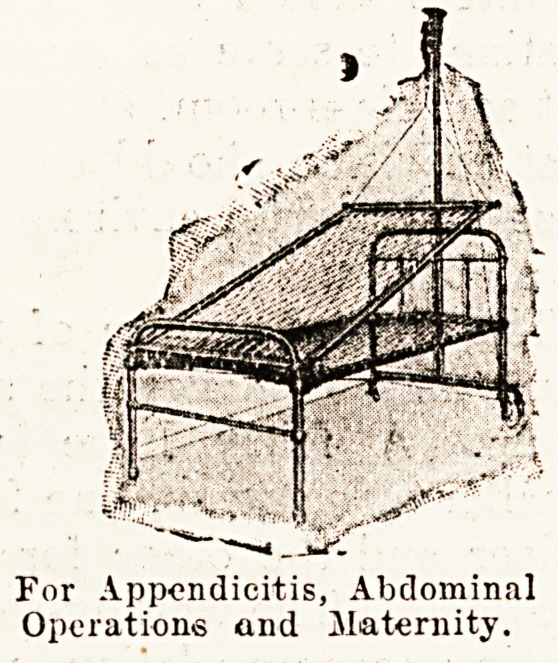


**Figure f3:**
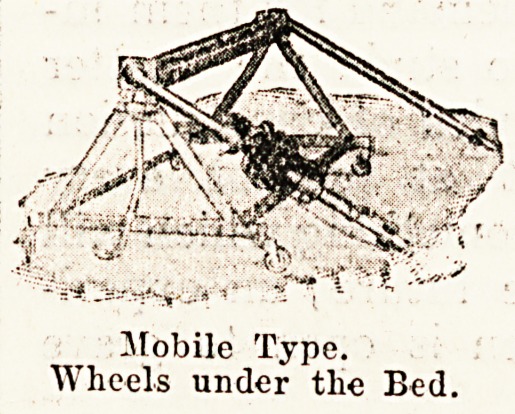


**Figure f4:**